# Insulin-like growth factor-1 receptor controls the function of CNS-resident macrophages and their contribution to neuroinflammation

**DOI:** 10.1186/s40478-023-01535-8

**Published:** 2023-03-08

**Authors:** Daniela C. Ivan, Kristina Carolin Berve, Sabrina Walthert, Gianni Monaco, Katharina Borst, Elisa Bouillet, Filipa Ferreira, Henry Lee, Jasmin Steudler, Thorsten Buch, Marco Prinz, Britta Engelhardt, Giuseppe Locatelli

**Affiliations:** 1 Theodor Kocher Institute, University of Bern, Freiestrasse 1, CH-3012 Bern, Switzerland; 2 Institute of Neuropathology, University of Freiburg, Freiburg, Germany; 3 Institute of Laboratory Animal Science, University of Zurich, Zurich, Switzerland; 4 Center for Basics in NeuroModulation (NeuroModulBasics), Faculty of Medicine, University of Freiburg, Freiburg, Germany; 5 Signalling Research Centres BIOSS and CIBSS, University of Freiburg, Freiburg, Germany

**Keywords:** IGF-1R, IGF-1, Microglia, Border associated macrophages, Experimental autoimmune encephalomyelitis, Multiple sclerosis

## Abstract

Signaling by insulin-like growth factor-1 (IGF-1) is essential for the development of the central nervous system (CNS) and regulates neuronal survival and myelination in the adult CNS. In neuroinflammatory conditions including multiple sclerosis (MS) and its animal model experimental autoimmune encephalomyelitis (EAE), IGF-1 can regulate cellular survival and activation in a context-dependent and cell-specific manner. Notwithstanding its importance, the functional outcome of IGF-1 signaling in microglia/macrophages, which maintain CNS homeostasis and regulate neuroinflammation, remains undefined. As a result, contradictory reports on the disease-ameliorating efficacy of IGF-1 are difficult to interpret, together precluding its potential use as a therapeutic agent. To fill this gap, we here investigated the role of IGF-1 signaling in CNS-resident microglia and border associated macrophages (BAMs) by conditional genetic deletion of the receptor *Igf1r* in these cell types. Using a series of techniques including histology, bulk RNA sequencing, flow cytometry and intravital imaging, we show that absence of IGF-1R significantly impacted the morphology of both BAMs and microglia. RNA analysis revealed minor changes in microglia. In BAMs however, we detected an upregulation of functional pathways associated with cellular activation and a decreased expression of adhesion molecules. Notably, genetic deletion of *Igf1r* from CNS-resident macrophages led to a significant weight gain in mice, suggesting that absence of IGF-1R from CNS-resident myeloid cells indirectly impacts the somatotropic axis. Lastly, we observed a more severe EAE disease course upon *Igf1r* genetic ablation, thus highlighting an important immunomodulatory role of this signaling pathway in BAMs/microglia. Taken together, our work shows that IGF-1R signaling in CNS-resident macrophages regulates the morphology and transcriptome of these cells while significantly decreasing the severity of autoimmune CNS inflammation.

**Supplementary Information:**

The online version contains supplementary material available at 10.1186/s40478-023-01535-8.

## Introduction

Microglia and border-associated macrophages (BAMs) compose the phagocytic immune resident compartment of the central nervous system (CNS) and inhabit the tissue parenchyma as well as the barrier-containing interfaces such as the choroid plexus, the leptomeninges and the perivascular spaces [25]. While the physiological and the pathological role of microglia has been extensively studied [31, 74, 77, 90], the contribution of BAMs to CNS homeostasis has only recently been investigated, thanks to technical advancements allowing to study these cells separately from short-lived bone marrow-derived myeloid cells [35, 95, 105]. These studies reveal a surprising heterogeneity of these cells within their distinct environments [95] and suggest a main role in immune surveillance, waste clearance, nutrient uptake and interaction with other patrolling cells [34]. Furthermore, experimental evidence indicates an important function of BAMs during pathological CNS conditions ranging from neurodegenerative diseases to ischemia [34] and including autoimmune neuroinflammation, in which early activation, local proliferation, augmented antigen presenting and immunomodulatory activities of BAMs have been described in different phases of disease development [45]. Nonetheless, the study of the complex role of these cells remains in its infancy [43].

Among their different pro- or anti-inflammatory actions [13], CNS-resident myeloid cells act as context-dependent immune-modulators by secreting cytokines and growth factors that support inflammation or tissue regeneration [64, 72, 98]. One of these molecules is Insulin-like growth factor 1 (IGF-1) [92] acting as a pro-remyelination [4, 61, 67, 104] and neuroprotective factor within the CNS [51] while being systemically produced by the liver as a hormone regulating the somatotropic axis. While an anti-inflammatory environment rich in IL-4 or cyclic adenosine monophosphate promotes the release of IGF-1 [8, 9, 92], a pro-inflammatory milieu (i.e. rich in IFN-γ) inhibits microglial IGF-1 production [92] and their ability to support oligodendrogenesis [7]. Beside its local production, IGF-1 levels in the CNS and in the cerebrospinal fluid (CSF) are also regulated by the import of peripheral IGF-1 across the CNS vasculature [102], a mechanism partially regulated by neuronal activity [69].

IGF-1 binds with high affinity to the IGF-1 receptor (IGF-1R) (M M Rechler and Nissley, 1985;[76], a tyrosine-kinase receptor mediating cellular growth, survival and sensing of external nutrient sources [14]. The molecule and its receptor are typically expressed in close proximity, indicating paracrine and autocrine signalling [70, 99]. In the CNS, IGF-1R is expressed by virtually all resident and invading inflammatory cells, with IGF-1 in turn being able to modulate the function of different inflammatory players [86]. In peripheral organs, signalling through IGF-1R showed an anti-inflammatory modulation of myeloid cell phenotypes in different disease models [32, 41, 50, 88]. Nonetheless, the specific actions controlled by IGF-1R signalling in microglia and BAMs remain unknown, together hindering our understanding of this pathway in CNS homeostasis and inflammation.

In this work, by employing tamoxifen-inducible genetic recombination in *CX3CR1-Cre*^*ERT2*^ × *IGF-1R*^*fl/fl*^ mice, we abrogated the expression of IGF-1R in CNS-resident macrophages. IGF-1R deletion did not affect the density of BAMs and microglia but led to altered cellular morphology in the brain and spinal cord. Bulk RNA sequencing of IGF-1R^KO^ and control CNS-resident myeloid cells suggested a higher basal activation in microglia and BAMs and showed a downregulation of genes and pathways involved in BAM migration in IGF-1R^KO^ mice. To assess whether these changes impacted the development of autoimmune CNS inflammation, we induced the neuroinflammatory model experimental autoimmune encephalomyelitis (EAE) and observed a consistently worsened clinical disease in mice carrying *Igf1r* deletion compared to controls.

In summary, our study describes for the first time the role of IGF-1 signalling in CNS-resident macrophages and shows that IGF-1R regulates the homeostatic phenotype and functions of these cells in turn influencing the development of autoimmune CNS inflammation.

## Material and methods

### Animals

Tamoxifen inducible *B6.129P2(C)-Cx3cr1*^*tm2.1(Cre(ERT2)Jung*^*/J (Cx3Cr1-cre*^*ERT2*^*)* mice were provided by Prof. Marco Prinz and crossed with C57BL/6j *IGF-1R floxed* mice [49] provided by Prof. Thorsten Buch and with *B6.Cg-Gt(ROSA)26Sor*^*tm14(CAG−tdTomato)Hze*^*/J* (*Ai14)* reporter mice [58] kindly provided by Dr. Urban Deutsch. Mice were kept under specific pathogen free conditions in individually ventilated cages and fed ad libitum. Animal experiments utilized female mice between the age of 6 and 12 weeks of age and were approved by the veterinary office of the Canton of Bern, Switzerland and were performed following the Swiss legislation on animal protection.

### Tamoxifen treatment

To induce Cre recombinase in six to ten weeks old *IGF-1R*^*KO−tdTomato*^ and *IGF-1R*^*WT−tdTomato*^ mice, we subcutaneously injected mice in four different places on the dorsal body area with a total of 200 μl of corn oil (50 μl/ spot) (Sigma-Aldrich, Cat.C8267) containing 4 mg Tamoxifen (Sigma-Aldrich, Cat. T5648) at day 0 and day 2. Tamoxifen was freshly dissolved prior to injection by thorough shaking for 2–3 h at room temperature.

### DNA isolation

Mice were sacrificed by isoflurane overdose and intracardially perfused with DPBS. Following CNS harvest, 20–30 mm thick brain and spinal cord pieces were incubated in 500 μl of lysis buffer (containing 1 M Tris hydrochloride (pH 7.5) (Sigma-Aldrich), 0.5 M Ethylenediaminetetraacetic acid (EDTA) (Sigma-Aldrich, Cat. 03,685), 20% Sodium dodecyl sulfate (SDS) (Sigma-Aldrich, Cat.436143), 5 M Sodium Chloride (NaCl) (Sigma-Aldrich, S9625), and distilled water) for 2–3 h or overnight at 55 °C under shaking conditions. Following 10 min centrifugation at 13′000 rpm, 4 °C, the supernatant was transferred to tubes filled with 500 μl isopropanol and centrifugated again for 10 min, at 13′000 rpm, 4 °C. Next, the supernatant was discarded, and the DNA pellet was washed with 70% ethanol. Following centrifugation (10 min, 13′000 rpm, 4 °C), the supernatant was discarded, and the remaining ethanol in the samples was allowed to evaporate under a sterile hood. The DNA pellet was dissolved in distilled water and the concentration and purity (ratio of absorbance at 260 and 280 nm) was assessed using a NanoDrop measuring system (Thermo Fischer Scientific). The samples were frozen at −20° C until further usage.

### Polymerase Chain Reaction (PCR)

DNA was amplified through polymerase chain reaction (PCR). Specifically, 1 μl of sample DNA was mixed with 12.54 μl distilled water, 4 μl of 5 × Green GoTaq flexi PCR buffer (Promega), 1.2 μl of 25 mM Magnesium Chloride (MgCl), 0.8 μl of 5 mM dNTPs, 0.13 μl *Igf1r* forward primer (CCC AAA CAG ACC ACC ACC A) (100 pmol/ μl), 0.13 μl *Igf1r* reverse primers (CTT CAG CTT TGC AGG TGC ACG) (100 pmol/ μl) and 0.2 μl GoTaq Flexi DNA Polymerase (Promega). The PCR reaction occurred under the following cycling conditions: initial denaturation (98 °C, 30 s), followed by 35 cycling times involving denaturation (98 °C, 30 s), annealing (70 °C, 30 s) and elongation (72 °C, 60 s). The reaction ended with a final elongation step at 72 °C, for 5 min. 10 μl of each sample were loaded on a 1% agarose gel and run for 50 min at 140 V. The expected band size for the *Igf1r* floxed is 1′200 bp, for *Igf1r* WT 1′100 bp and 600 bp for the *Igf1r* locus following genetic recombination.

### EAE induction

Mice were induced with active EAE six to ten weeks post Tamoxifen treatment (after replenishment of IGF-1R^+^ blood monocytes by natural turnover), as previously described [44]. Briefly, at day 0, mice were immunized with an emulsion of 200 μg MOG peptide (MOG_35-55_, Genscript, USA) and complete Freund´s adjuvant (Santa Cruz Biotechnology) containing Mycobacterium tuberculosis (Difco) (100 μl/mouse subcutaneously, in the left and right flank and at the tail base). Furthermore, 400 ng pertussis toxin (List Biological Laboratories, Campbell, CA, USA) diluted in sterile DPBS was administered intraperitoneally at day 0 and day 2.

The weight of the mice was recorded daily and the clinical disease course was assessed using a 0–3 score scale as described in [93]. Mice displaying a 3–5% weight loss but no other motor impairments received a score of 0 (weight-loss stage), animals showing a limp tail were scored as 0.5 (day of clinical onset), mice displaying a partial weakening of hind limbs received a score of 1 and animals presenting hind limb paraparesis/paraplegia were scored with 2 (symptomatic disease peak). Mice displaying hindlimb paralysis and forelimbs paraparesis were scored with 3 and met termination criteria. For each EAE group, the disease incidence as well as the area under the curve was calculated to assess the clinical disease evolution.

### Isolation of CNS resident and infiltrating myeloid cells and flow cytometry analysis/ cell sorting

Mice were sacrificed by isoflurane overdose, perfused with 20 ml ice-cold DPBS and the brain and spinal cords were harvested and processed separately for *CX3CR1-cre*^*ERT2*^×* IGF-1R floxed* mice. The tissue was finely cut/ homogenized in collagenase medium containing 0.4 mg/ml Collagenase VIII (Sigma, C2139) and 2 U/ml DNAse (Roche) and incubated afterwards in a 37 °C water bath, under shaking conditions, for 30 min. During this incubation time, the samples were gently resuspended every 10 min to help the homogenization of the tissue. The enzymatic reaction was stopped by sample incubation with 0.5 M EDTA (1:100) for 5 min at 37 °C. The samples were then transferred on ice and filtered through a 70 μm cell strainer (SPL Life Sciences, Cat. 93,070). Following 5 min centrifugation (1′500 rpm, 4 °C), the CNS pellets were resuspended in 13 ml of 33% Percoll (Ge Healthcare Biosciences, 17-0891-01) solution and centrifugated for 30 min at 4′000 rpm, 4 °C without breaks. This step allows the creation of a density gradient formed by a myelin ring at the surface, a middle layer containing CNS resident and infiltrating myeloid cells and a blood cell ring at the bottom of the tube. The middle layer containing the cells of interest was transferred to a new tube, filtered through 100 μm cell strainer and washed twice with DPBS. Following centrifugation (5 min, 1′500 rpm, 4 °C), and supernatant was discarded and the cells were prepared for antibody staining. For this step, the cells were initially incubated with anti-CD16/32 (homemade solution) for 15 min on ice to block the FC-receptors and after DPBS wash, the cells were incubated for 30 min at 4 °C, in dark condition, with the following antibodies diluted in FACS buffer (containing 1% FCS (SeraGlob, S11500), and DPBS): CD11b (FITC or PerCP Biolegend, Clone M1/70, Cat.101230), CD45 (LSBio, Lot 62,579), CD44 (Biolegend, Brilliant Violet 605, clone IM7, Cat. 103,047), CD206 (Biolegend, Brilliant Violet 711, Clone C068C2, Cat. 141,727), Ly6C (Biolegend, Alexa Fluor 700, clone HK1.4, Cat.128024), Ly6G (Biolegend, APC/Cy7, Clone 1A8, Cat.127624), MHC-II (Biolegend, Brilliant Violet 711, Clone C068C2, Cat.141727), Fixable viability dye eFluor 506 (Invitrogen, Rockford, IL, USA, cat. 65–0866-14). The compensations were performed using unstained cells and single antibody stainings and the samples were acquired using an Attune NxT cytometer (Thermo Fisher Scientific). For data analysis, we used the software FlowJo (version 10, Ashland, OR, USA).

The following gating strategy was used to investigate the phenotype of different myeloid cell populations: after identifying cells of interest based on forward and side scatter, we selected single cells (FSC-H vs FSC-A). We performed the analysis on two distinct live cell populations: CD45^high^CD11b^+^ and CD45^intermediate^CD11b^+^ cells. Within the CD45^high^CD11b^+^ population, we selected Tomato + cells, thus identifying CD45^high^CD11b^+^Tomato^+^ BAMs and CD45^high^CD11b^+^Tomato^neg^ cells (CNS infiltrating blood-derived myeloid cells). Finally, the CD45^high^CD11b^+^Tomato^neg^Ly6G^neg^Ly6C^+^ population represented CNS infiltrating monocyte-derived macrophages. CNS resident microglial cell population was identified based on CD45^intermediate^CD11b^+^Tomato^+^ expression. In these populations we analyzed the expression of MHC-II, CD206 and CD44. As readout, we displayed the percentage or absolute number of tdTomato positive cells (BAMs and microglia) or Ly6C + cells (infiltrating monocyte-derived macrophages) and the relative mean fluorescence intensity (MFI) calculated by subtracting the MFI of antibody staining from the MFI of isotype control staining.

For cell sorting, cells were incubated with blocking anti-CD16/32 ab (eBioscience) for 15 min on ice and after DPBS wash, cells were incubated for 45 min at 4 °C, in dark condition, with the following antibodies diluted in FACS buffer (containing 1% FCS (SeraGlob, S11500), and DPBS): CD3 (APC-Cy7 Biolegend), CD19 (APC-Cy7 Biolegend), NK1.1 (APC-Cy7 Biolegend), Ly6C (APC-Cy7 Biolegend), cell viability dye eFluor 506), CD11b (BV605 Biolegend), CD45 (BV421 Biolegend), CD206 (FITC Biolegend), P2ry12 (Biolegend, APC). Samples were sorted into RNAprotect buffer using an MoFlow Astrios (Beckman Coulter).

### CNS isolation, decalcification and cryostat sections

Mice were deeply anesthetized and transcardially perfused initially with DPBS (Gibco, Paisley, UK) and then with 4% paraformaldehyde (PFA, Merk Darmstadt, Germany). Entire skulls as well as spinal columns were isolated and post-fixed in 4% PFA overnight at 4 °C. Following PBS wash, organs were decalcified for 7 to 10 days at 4 °C in 14% EDTA solution (Sigma-Aldrich, Cat. E5135) (prepared in osmose water and adjusted to a pH of 7.8 – 8) (with a change of EDTA every second day) [42]. At the end of the decalcification procedure, the softness of the bones was tested by gentle touching with a pair of tweezers. Brain and spinal cord tissues were washed with PBS, immersed for 3 days in 30% sucrose (Sigma-Aldrich, St. Louis, MO, USA) and frozen at -80 °C in O.C.T (Tissue-Tek). 40 µm sagittal brain section and 25 µm spinal cord longitudinal sections were cut using a cryostat (HM550, Thermo Fisher).

### Immunofluorescence stainings of CNS tissue

IGF-1R, GFAP and myelin basic protein (MBP) immunofluorescence stainings were performed on cryostat-cut decalcified spinal cord sections as follows: for IGF-1R and GFAP stainings, the tissue was fixed with 100% ice cold acetone at −20 °C for 10 min and dried before being reconstituted in homemade 1xTris-Buffered Saline (TBS). Alternatively, for MBP staining, sections were fixed with 100% ice cold methanol at −20 °C for 10 min and immediately washed for a total of 30 min in TBS. Unspecific antibody binding was blocked for 2 h at room temperature (RT) either with 10% goat serum containing 0.1% Triton (Sigma-Aldrich, St. Louis, MO, USA) diluted in TBS (for IGF-1R and GFAP stainings) or with 5% BSA containing 0.3% Triton diluted in TBS (for MBP staining). Sections were then incubated at 4 °C overnight with the following primary antibodies: polyclonal rabbit anti-IGF1R (phospho-Y1161, Abcam, ab39398, 1:100), polyclonal rabbit anti-GFAP (Dako, Z0334, 1:100) diluted in 2% goat serum in TBS containing 0.1% Triton and a monoclonal rat anti-MBP (aa82-87, BioRad, MCA409S, 1:100) diluted in 1% BSA in TBS containing 0.3% Triton. After rinsing 3 × 10 min with TBS, spinal cord sections were incubated for 2 h at RT with the following secondary antibodies: Alexa Fluor 647 goat anti-rabbit (Invitrogen, A32733), Alexa Fluor 488 goat anti-rabbit (Invitrogen, A11008) diluted (1:200) in 2% goat serum in TBS and Alexa Fluor 488 goat anti-rat (Invitrogen, A-11006) (1:200) diluted in 1% BSA in TBS. Following a 3 × 10 min wash with TBS, slices were incubated with DAPI (1:5000 in TBS, 1 mg/ml stock, AppliChem, Darmstadt, Germany) for 10 min at RT, after which they were mounted with Mowiol 4–88 solution (Sigma-Aldrich, St Louis, MO, USA) and left to dry.

For IGF1-R staining, Z-stack images of CNS sections were acquired using a LSM800 confocal microscope (Zeiss) with 25 × and 40 × objectives. Images of GFAP and MBP stainings were acquired using a fluorescence microscope Nikon Eclipse E600 with 10 × and 20 × objectives. All images were analyzed using the software Fiji (National Institute of Health, Bethesda, MD, USA).

### Analysis of inflammatory lesions, density and morphology of microglia/BAMs

To assess the density and size of inflammatory lesions, spinal cord sections from *IGF-1R*^*KO−tdTomato*^ (*n* = 5) and *IGF-1R*^*WT−tdTomato*^ mice (*n* = 4) were stained with DAPI and unphysiological accumulations of DAPI + structures (as indicators for immune cell infiltration) were manually selected via Fiji. Single lesion area (μm^2^) was calculated and number of well-defined single lesions over the entire tissue area was assessed (cm^2^). To define the degree of demyelination, white matter areas of spinal cord tissue sections were selected and MBP staining intensity assessed via Fiji. To assess the density and morphology of BAMs and microglial cells in healthy *IGF-1R*^*KO−tdTomato*^ (*n* = 4) and *IGF-1R*^*WT−tdTomato*^ mice (*n* = 4), we used the Fiji software. From each mouse, Z-stack images were acquired from brain (cortex, midbrain and cerebellum) and spinal cord (cervical, thoracic, lumbar/sacral) regions. Per region, we acquired three representative images. To analyze the density of microglia and leptomeningeal and perivascular BAMs, we used maximum intensity projection of confocal z-stack images acquired with a 25 × objective. pvMs were defined as elongated Tomato + cells delineating blood vessels. Following image conversion to binary mode, we selected three to four regions of interest per image, used a threshold and a cell circularity range tested to cover most cells in the acquired images, and used the ´count cell` function to assess the number of cells/mm^2^. We further assessed the morphology of individual microglia within the brain and spinal cord of WT and KO mice (confocal microscopy) as well as the morphology of leptomeningeal BAMs (two-photon intravital imaging). For the description of leptomeningeal BAMs, two-photon movies from the intact spinal cord were favored over tissue sections to better preserve the physiological morphology of these cells in their CSF-filled microenvironment. We used three different methods: Sholl analysis [60, 66, 85], skeleton analysis and fractal—lacunarity analyses [65, 106]. Each was performed on maximum intensity projection of confocal z-stack images acquired with a 40 × objective. Prior to morphological analyses, we selected images in a blinded manner. Each image was converted to binary mode and the ´unsharp mask filter´ as well as the ´noise-despecle´ function were applied in Fiji to detect as many cell branches as possible. When needed, following image thresholding, we manually reconstructed the branches lost during this procedure.

Sholl Analysis: after manually indicating the largest Sholl radius from the center of each soma, we defined the starting radius at 5 μm and the radius step size at 2 μm and selected the Sholl `intersections` method. As outcome for cell morphological analysis, we used the following parameters: the maximum number of intersections per cell irrespective of the radius (Max Inters.), the critical value/the distance from the soma where the maximum number of intersections occurred (Max.Inters. Radius) and the maximum radius where the longest branch occurred (Ending Radius) [66]. Since Sholl analysis provides only information related to cell branches, we also manually measured the soma size (area) of each analyzed cell. Skeleton analysis: on binary images, we used the `analyze skeleton 2D/3D´ function in Fiji and chose the following parameters: number of branches/ cells, maximum branch length, number of endpoints/ cell and number of junctions/ cells. Additionally, to obtain an indication about the cell complexity, we calculated the summed branch length for each cell.

Lastly, we used Fractal and Lacunarity analyses as a more in-depth method to determine the complex geometry of individual cells as well as their shape/elongation. In biology, fractal analysis measures the fractal characteristics of a data set (the degree to which a cell fills the occupied space) [47]. Complementary, lacunarity analysis assesses the gaps between cell branches and the heterogeneity of their distribution. Very ramified cells display low lacunarity whereas high lacunarity indicates poor cellular branching. From these two analyses, we extracted the following parameters: cell density, span ratio and circularity (as a measure of cell shape/elongation), fractal dimension, lacunarity and maximum span across convex hull (as measures of cell complexity). The number of individual microglia analyzed was the following: WT (4 mice: brain *n* = 71 cells, spinal cord *n* = 66 cells), KO (4 mice, brain *n* = 83 cells, spinal cord *n* = 76 cells). The number of individual leptomeningeal BAMs analyzed was the following: WT (3 mice: *n* = 90 cells). KO (3 mice: *n* = 77 cells).

### Two-photon intravital microscopy

The cervical spinal cord was exposed by laminectomy as previously described [38]. Briefly, animals were intubated by insertion of a canula in the trachea providing oxygen and isoflurane through a Minivent system (Harvard apparatus). Laminectomy (C2-5) was performed and spinal column fixed at C1 and C6 in a stereotactic frame. Body temperature and heart rate were recorded and stored electronically. Imaging was performed via 2-photon (LaVision TrimScope II microscope, Spectra-Physics laser). Mouse respiration via MiniVent was synchronized to picture acquisition through a triggering device (TrigViFo) reducing imaging artifacts [97]. Through an additional toolkit (VivoFollow II) [97], this synchronization allowed real-time distortion correction. Tissue displacement was automatically corrected by objective and stage adjustment through a Python script (VivoFollow I) [96]. Single images, z-stacks and videos were acquired with ImSpector software (LaVision). Raw images were processed with Fiji [82] and saved as TIF.

### RNA extraction, library preparation and sequencing

Microglia and BAMs were sorted from the brain of *IGF-1R*^*KO−tdTomato*^ and *IGF-1R*^*WT−tdTomato*^ mice and processed for RNA sequencing. RNA extraction, library preparation and sequencing were performed at the Genomics Core Facility “KFB—Center of Excellence for Fluorescent Bioanalytics”. Total RNA was extracted from sorted cells stabilized in RNAprotect buffer according to the RNeasy Plus Micro Kit protocol (QIAGEN, Hilden, Germany). In brief, cells were stored and shipped in buffer RNAprotect at 2–8 °C. After pelleting by centrifugation for 5 min at 5′000 × g, the RNAprotect was replaced by 350 µl buffer RLT Plus and the samples were homogenized by vortexing for 30 s. Genomic DNA contamination was removed by using gDNA Eliminator spin columns. One volume of 70% ethanol was added and the samples were applied to RNeasy MinElute spin columns followed by several wash steps. Total RNA was eluted in 12 μl of nuclease free water. Purity and integrity were assessed on the Agilent 2100 Bioanalyzer with the RNA 6000 Pico LabChip reagent set (Agilent, Palo Alto, CA, USA). The SMARTer Ultra Low Input RNA Kit for Sequencing v4 (Clontech Laboratories, Inc., Mountain View, CA, USA) was used to generate first strand cDNA from 1 ng total-RNA. Double stranded cDNA was amplified by LD PCR (12 cycles) and purified via magnetic bead clean-up. Library preparation was carried out as described in the Illumina Nextera XT Sample Preparation Guide (Illumina, Inc., San Diego, CA, USA). 150 pg of input cDNA were tagged and fragmented by the Nextera XT transposome. The products were purified and amplified via a limited-cycle PCR program to generate multiplexed sequencing libraries. For the PCR step 1:5 dilutions of the unique dual indexing (i7 and I5) adapters were used. The libraries were quantified using the KAPA Library Quantification Kit—Illumina/ABI Prism User Guide (Roche Sequencing Solutions, Inc., Pleasanton, CA, USA). Equimolar amounts of each library were sequenced on a NextSeq 500 instrument controlled by the NextSeq Control Software (NCS) v2.2.0, using two 150 Cycles High Output Kits with the dual index, single-read (SR) run parameters. Image analysis and base calling were done by the Real Time Analysis Software (RTA) v2.4.11. The resulting.bcl files were converted into.fastq files with the bcl2fastq v2.18 software.

### Transcriptomics analysis

Bulk RNA-seq data was aligned with STAR v2.7.8a [23] to the mouse reference genome Gencode M26 [40]. Genes with sum count expression less than 5 were filtered out. Differential expression was performed using the limma trend method [78] on the log2 counts per million (CPM) values. Heatmaps were generated with the R packages pheatmap; enrichment analysis was performed with the enrichGO function from the R package ClusterProfiler and using the Gene Ontology database.

### Statistics

Statistical analysis was performed using GraphPad Prism 9 software (La Jolla, CA, USA).

All data was assessed for normality distribution. Shapiro–Wilk test was used to assess the normality for small sample sizes (< 50) while Kolmogorov Smirnov test was used for larger sample size (> 50). Data that was not normally distributed was analyzed with a non-parametric Mann Whitney U test: body weight differences before and after tamoxifen treatment, quantification of IGF-1R deletion in CNS resident perivascular BAMs, morphological analysis of microglia and BAMs, EAE AUC and incidence. We further used unpaired t test with Welch`s correction for the following sets of data: quantification of IGF-1R deletion in meningeal BAMs and microglial cells, density of CNS-resident myeloid cells following genetic ablation of IGF-1R (brain BAMs, spinal cord microglia and BAMs), morphological analysis of brain microglia (cell density, maximum branch length), spinal cord microglia (cell density, maximum span across hull, fractal dimension, number of branches, maximum branch length), spinal cord BAMs (fractal dimension, lacunarity), integrin expression and flow cytometry characterization of cell number and phenotype during EAE, number of inflammatory lesions per area, area of individual lesions during EAE, MBP staining intensity and GFAP + cells/mm^2^. Two-way ANOVA with Tukey`s multiple comparisons test was used for assessing the EAE severity (score differences) between *IGF-1R*^*KO−tdTomato*^ and *IGF-1R*^*WT−tdTomato*^ mice. All values are presented as mean ± standard error of the mean (SEM). Asterisks indicate significant differences (∗ *p* < 0.05, ∗  ∗ *p* < 0.01 and ∗  ∗  ∗ *p* < 0.001, ∗  ∗  ∗  ∗ *p* < 0.0001).

## Results

### Genetic deletion of *Igf1r* in CNS-resident microglia and BAMs

**Fig. 1:**
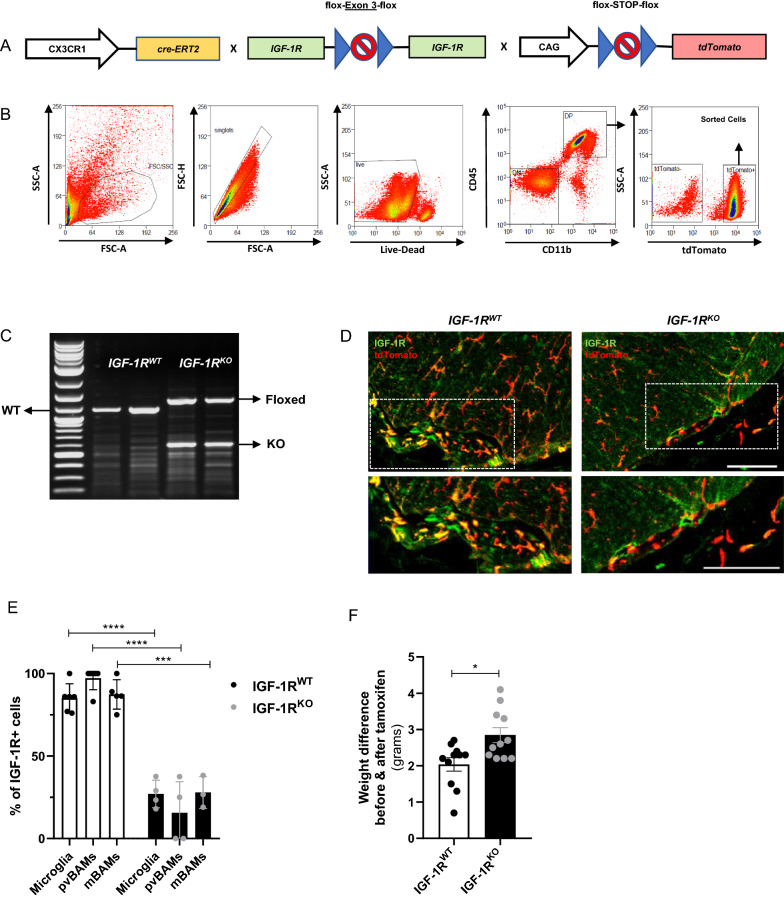
Characterization of *IGF-1R*^*KO*^ mouse model. **A** Diagram illustrating the genetic make-up of the *CX3CR1-cre*^*ERT2*^* x IGF-1R floxed x Ai14* mouse model. **B** Gating strategy illustrating the sorting of tdTomato^+^ CD45 + CD11b + myeloid cells from the CNS of tamoxifen-treated *IGF-1R*^*KO−tdTomato*^ and *IGF1R*^*WT−tdTomato*^ mice. **C** DNA from sorted cells was tested by PCR using specific primers identifying the WT (1′100 bp), floxed (1′200 bp) or recombined (600 bp) *Igf1r* locus (*n* = 2). **D** Representative confocal pictures and **E** quantification of IGF-1R expression in Tomato + cells following staining with IGF-1R-specific antibody in CNS sections from *IGF-1R*^*KO−tdTomato*^ (n = 3) and *IGF-1R*^*WT−tdTomato*^ mice (*n* = 4): unpaired T-test with Welch´s correction, *p* < 0.0001 (microglia), *p* < 0.001 (meningeal BAMs) and Mann Whitney U test *p* = 0.0048 (perivascular BAMS) **F)** Bar graph shows difference in weight before and after tamoxifen treatment measured in *IGF-1R*^*KO−tdTomato*^ (*n* = 11) and *IGF-1R*^*WT−tdTomato*^ mice (*n* = 11) from 3 different experiments (Mann Whitney U test, *p* = 0.031). All values are presented as mean ± SEM. Asterisks indicate significant differences (∗ *p* < 0.05, ∗  ∗ *p* < 0.01 and ∗  ∗  ∗ *p* < 0.001, ∗  ∗  ∗  ∗ *p* < 0.0001)

IGF-1 signalling is a main regulator of metabolism, proliferation and survival in numerous cell types, but its function in CNS-resident macrophages remains undetermined. To shed light on this unclear role, we genetically deleted the receptor *Igf1r* from CX3CR1-expressing myeloid cells by crossing a tamoxifen-inducible mouse model, *CX3CR1-cre*^*ERT2*^ [105]*,* with a transgenic mouse carrying a loxP-flanked 3rd exon in the *Igf1r* gene *(IGF-1R floxed)* [49]. In the resulting *CX3CR1-cre*^*ERT2*^ × *IGF-1R floxed* model, recombination is achieved by subcutaneous administration of tamoxifen [19] followed by a waiting time of 5 weeks during which short-lived bone marrow-derived CX3CR1^+^ monocytes are replenished through natural turnover by new IGF1R^+^ cells. Long-lived BAMs and microglia instead maintain their IGF-1R^KO^ status. To distinguish in situ CNS-resident macrophages from short-lived bone marrow-derived myeloid cells, mice were further crossed to a cre-inducible tdTomato reporter *Ai14* mouse [58] allowing expression of the fluorescent molecule tdTomato in BAMs/microglia. Taken together, we created the new animal model *CX3CR1-cre*^*ERT2*^×* IGF-1R floxed x Ai14* (henceforth *IGF-1R*^*KO−tdTomato*^) and its cre^+^ control line *CX3CR1-cre*^*ERT2*^ × *Ai14* (henceforth *IGF-1R*^*WT−tdTomato*^) (Fig. 1A).

To assess whether genetic recombination occurred in CNS-resident macrophages of *IGF-1R*^*KO−tdTomato*^ mice, we isolated CD45^high^CD11b^+^ (BAMs) and CD45^intermediate^CD11b^+^ (microglial) cells from the CNS of tamoxifen-treated *IGF-1R*^*KO−tdTomato*^ and *IGF-1R*^*WT−tdTomato*^ mice (Fig. 1b). PCR analysis of sorted cells (Fig. 1c) showed that genetic recombination occurred in *IGF-1R*^*KO−tdTomato*^ but not in *IGF-1R*^*WT−tdTomato*^ mice. However, since recombination appeared incomplete (Fig. 1c), we quantified the efficiency of receptor deletion at a protein level on decalcified CNS sections from *IGF-1R*^*KO−tdTomato*^ and *IGF-1R*^*WT−tdTomato*^ control mice. Confocal analysis of sections stained with IGF-1R-specific antibodies revealed receptor expression in 27% of microglial cells and in circa 21% of BAMs in *IGF-1R*^*KO−tdTomato*^ mice, compared to 85% of microglia and 92% of BAMs in *IGF-1R*^*WT−tdTomato*^ mice, collectively showing a deletion efficiency of approx. 68% for microglia and circa 75% for BAMs (Fig. 1D, E).

Considering the key systemic role of IGF-1 in promoting growth [62], we further assessed the weight of mice before tamoxifen administration and five weeks later. Surprisingly, we found that *IGF-1R*^*KO−tdTomato*^ mice gained significantly more weight compared to *IGF1R*^*WT−tdTomato*^ mice (Fig. 1F), thus suggesting that the induced macrophage-specific IGF-1R deletion can indirectly impact the somatotropic axis of treated mice.

Taken together, tamoxifen administration in *IGF-1R*^*KO−tdTomato*^ mice resulted in IGF-1R ablation from the majority of microglia/BAMs in the mouse CNS and led to a significantly higher weight gain in *IGF-1R*^*KO−tdTomato*^ compared to *IGF-1R*^*WT−tdTomato*^ mice.

### IGF-1R deletion leads to morphological changes in BAMs and microglia

**Fig. 2:**
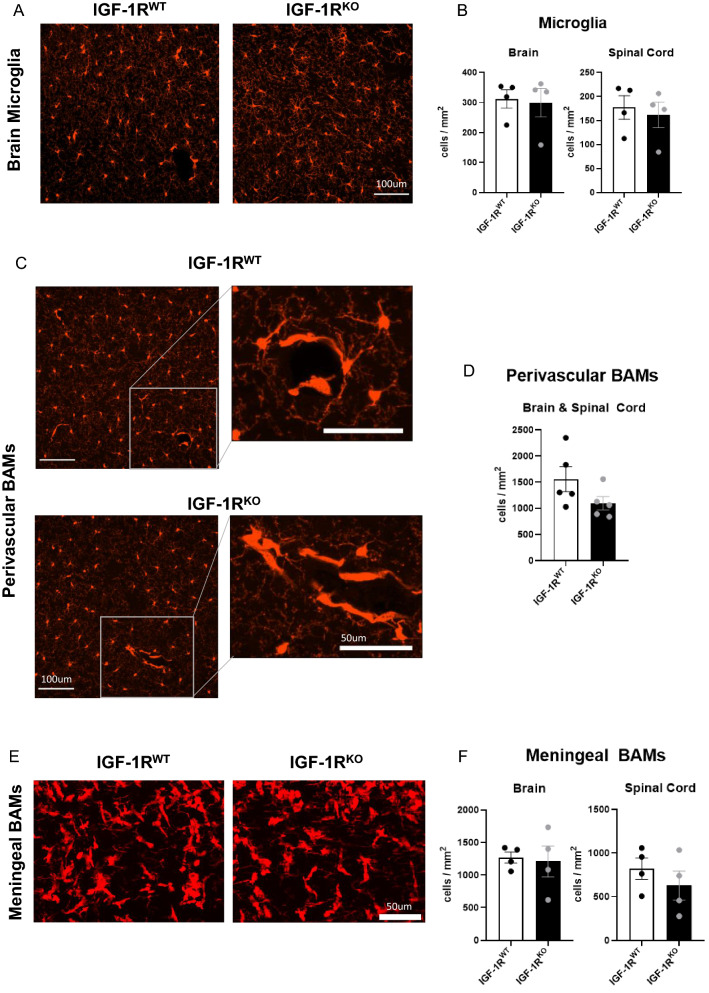
The density of CNS-resident myeloid cells following *Igf-1r* genetic ablation**. A** Representative pictures (scale bar 100 μm) and **B** bar graph illustrating the density of Tomato + microglia in brain and spinal cord sections from *IGF-1R*^*WT−tdTomato*^ mice (*n* = 4, number of cells analysed: microglia brain *n* = 49, microglia spinal cord *n* = 28) and *IGF-1R*^*KO−tdTomato*^ (*n* = 4 mice, number of cells analysed: microglia brain *n* = 45, Microglia spinal cord *n* = 42). **C** Representative pictures of Tomato + perivascular BAMs (pvBAMs) in the brain (scale bar zoom-out image: 100 μm, scale bar zoom-in image: 50 μm) and **D** bar graph illustrating the density of pvBAMs in brain and spinal cord sections (pooled *p* = 0.0048 together) from *IGF-1R*^*WT−tdTomato*^ (*n* = 4, number of cells analysed: 31) and *IGF-1R*^*KO−tdTomato*^ (*n* = 4 mice, number of cells analysed: 33). **E** Representative pictures and **F** bar graph illustrating the density of Tomato + meningeal BAMs (mBAMs) in brain and spinal cord sections from *IGF-1R*^*WT−tdTomato*^ (n = 4, number of cells analysed: mBAMs brain n = 31, mBAMs spinal cord *n* = 13) and *IGF-1R*^*KO−tdTomato*^ (*n* = 4 mice, number of cells analysed: BAMs brain *n* = 26, BAMs spinal cord *n* = 20). No statistically significant differences were observed between either group. Statistical analysis performed by unpaired t test with Welch’s correction: brain microglia, *p* = 0.833, spinal cord microglia *p* = 0.684, pvBAMs *p* = 136, brain mBAMs *p* = 0.828 and spinal cord mBAMs *p* = 0.3878

Since IGF-1 signalling regulates cell survival and proliferation [39], we assessed whether IGF-1R deletion affected the numbers of microglia as well as perivascular and meningeal BAMs, but found no significant change in their density in either the brain or the spinal cord of *IGF-1R*^*KO−tdTomato*^ and *IGF-1R*^*WT−tdTomato*^ mice (Fig. 2A, B – microglia, Fig. 2C, D – perivascular BAMs, Fig. 2E, F – meningeal BAMs).

**Fig. 3:**
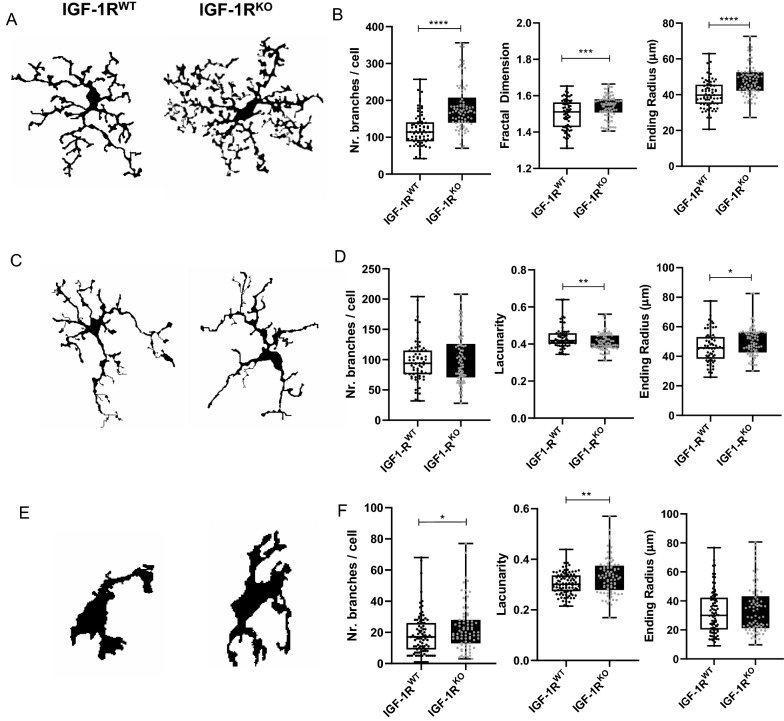
Morphological characterization of CNS-resident myeloid cells following *Igf-1r* genetic ablation. To assess the morphology of CNS resident microglia (brain, spinal cord) and leptomeningeal BAMs in *IGF-1R*^*KO−tdTomato*^ (n = 4 mice) compared to *IGF-1R*^*WT−tdTomato*^ (*n* = 4 mice), we conducted three sets of analyses (Sholl analysis, skeletal analysis and fractal-lacunarity analysis – described in the method section). **A** Representative pictures of microglia from the brain of *IGF-1R*^*KO−tdTomato*^ and *IGF-1R*^*WT−tdTomato*^ mice and **B** quantification of number of branches per cell (*p* = 0.0001), fractal dimension (*p* = 0.0006), ending radius (*p* < 0.0001) of brain microglia (unpaired t test with Welch’s correction, approximative n° of cells analysed per group: *IGF-1R*^*KO−tdTomato*^ (*n* = 84) *IGF-1R*^*WT−tdTomato*^ (*n* = 71)). **C** Representative pictures of microglia morphology from the spinal cord of *IGF-1R*^*KO−tdTomato*^ and *IGF-1R*^*WT−tdTomato*^ mice and **D** quantification of number of branches per cell (p = 0.498), fractal dimension (*p* = 0.406), ending radius (*p* = 0.034) of spinal cord microglia (unpaired t test with Welch’s correction, approximative n° of cells analysed per group: *IGF-1R*^*KO−tdTomato*^ (*n* = 75) *IGF-1R*^*WT−tdTomato*^ (*n* = 66)). **E** Representative pictures of BAMs from the spinal cord leptomeninges of *IGF-1R*^*KO−tdTomato*^ and *IGF-1R*^*WT−tdTomato*^ mice and **F** quantification number of branches per cell (*p* = 0.034), fractal dimension (*p* = 0.760), ending radius (*p* = 0.049) of spinal cord BAMs (unpaired t test with Welch’s correction, approximative n. of cells analysed per group: *IGF-1R*^*KO−tdTomato*^ (*n* = 78) *IGF-1R*^*WT−tdTomato*^ (*n* = 91)). All values are presented as mean ± SEM. Asterisks indicate significant differences (∗ *p* < 0.05, ∗  ∗ *p* < 0.01 and ∗  ∗  ∗ *p* < 0.001, ∗  ∗  ∗  ∗ *p* < 0.0001)

Considering that microglia functionality and activation are directly related to dynamic changes in their cell shape, size and ramifications [21, 36, 47], we analyzed the morphology of individual microglial cells on tissue sections from the brain and spinal cord of healthy *IGF-1R*^*KO−tdTomato*^ and *IGF-1R*^*WT−tdTomato*^ mice by Sholl analysis [60, 66, 85], skeleton analysis [2] and fractal analysis [65, 106]. Notably, brain microglia from *IGF-1R*^*KO−tdTomato*^ mice displayed significantly increased ramifications (increased number of branches, higher total cell branch length and maximum branch length, more junctions and endpoints of contact) and a more complex morphology (defined by fractal dimension and the maximum distance between branches in a convex hull) (Fig. 3A, B, Additional file 1: Fig. 1A). In parallel, in the spinal cord of *IGF-1R*^*KO−tdTomato*^ mice, microglia displayed lower lacunarity as well as an increased cell density and ending radius (defined by the maximum Sholl radius) (Fig. 3C, D, Additional file 1: Fig. 1B). Next, we assessed whether the morphology of BAMs would also be affected by the absence of IGF-1R. Given their location in confined perivascular spaces as well as their typical elongated shape stretching along the vessel, we refrained from analysing differences in pvMs morphology. However, we imaged by 2-photon microscopy the cervical spinal cords of anesthetized *IGF-1R*^*KO−tdTomato*^ and *IGF-1R*^*WT−tdTomato*^ mice and analyzed the morphology of leptomeningeal BAMs in vivo. Despite similar cell size and shape, BAMs from *IGF-1R*^*KO−tdTomato*^ mice displayed higher lacunarity, more branches and an increased number of endpoints and branch junctions compared to control mice (Fig. 3E, F, Additional file 1: Fig. 1C).

Together, these results indicate that the lack of IGF-1R in CNS resident myeloid cells induced significant changes in the morphology of these cells, in turn suggesting that IGF-1 signalling regulates the overall phenotype of BAMs and microglia during homeostasis.

### RNA analysis reveals distinct transcriptomic changes in microglia and BAMs upon IGF-1R deletion

**Fig. 4:**
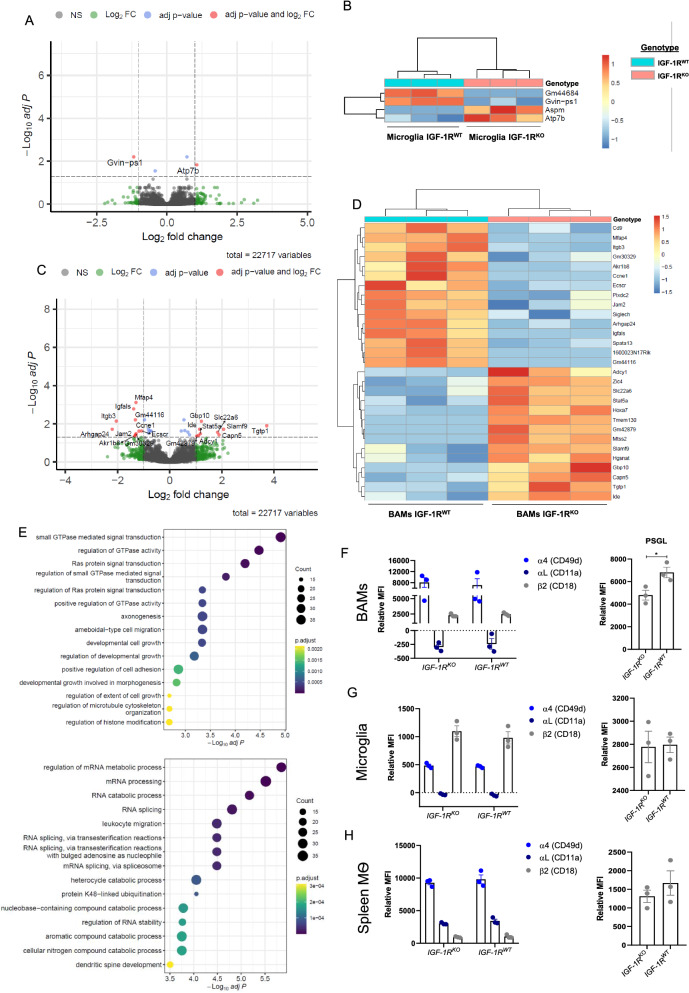
Transcriptomic analysis and expression of adhesion molecules in microglia and BAMs following genetic ablation of *Igf1r. A* Volcano plot and **B** heatmap showing the differential expression analysis of microglia from *IGF-1R*^*KO−tdTomato*^ and *IGF-1R*^*WT−tdTomato*^ mice sorted from brain. **C** Volcano plot and **D** heatmap showing the differential expression analysis of BAMs sorted from the brain of *IGF-1R*^*KO−tdTomato*^ and *IGF-1R*^*WT−tdTomato*^ mice **E** Functional enrichment analysis of BAMs from *IGF-1R*^*KO−tdTomato*^ and *IGF-1R*^*WT−tdTomato*^ mice using the Biological Process pathways from the Gene Ontology database, above: pathways upregulated in *IGF-1R*^*KO−tdTomato*^ mice, below: pathways downregulated in *IGF-1R*^*KO−tdTomato*^ mice. **F–H** Flow cytometry analysis of expression of α4 (CD49d), αL (CD11a) and β2 (CD18) integrins and PSGL-1 in BAMs, microglia and splenic macrophages from *IGF-1R*^*KO−tdTomato*^ (*n* = 3) and *IGF-1R*^*WT−tdTomato*^ (*n* = 3) mice. Data represented as mean ± SEM. Statistical analysis was performed by using unpaired t test with Welch´s correction, *p < 0.05: BAMs (α4: *p* = 0.757; β2: *p* = 0.629, PSGL-1: *p* = 0.034), Microglia (α4: *p* = 0.591; β2: *p* = 0.466, PSGL-1: *p* = 0.907), splenic macrophages (α4: *p* = 0.539; αL: *p* = 0.281; β2: *p* = 0.629, PSGL-1: *p* = 0.402)

To dissect the functional impact of IGF-1R deletion in CNS-resident phagocytes, we isolated BAMs and microglial cells from the brain and meninges of *IGF-1R*^*KO−tdTomato*^ and *IGF-1R*^*WT−tdTomato*^ control mice (Additional file 2: Fig. 2A) and performed bulk RNA sequencing analysis. Transcriptomic changes were observed both in *IGF-1R*^*KO−tdTomato*^ microglia and BAMs compared to their *IGF-1R*^*WT*^ counterparts from *IGF1R*^*WT−tdTomato*^ mice (Additional file 2: Fig. 2B). Surprisingly, given the substantial changes in cellular morphology observed in *IGF-1R*^*KO−tdTomato*^ mice, microglial transcriptome was only partially modified by *Igf1r* deletion (Fig. 4A). Within these cells we found the ATPase 2 (*Atp7b)* gene, which is involved in the export of excess intracellular copper [110], to be the most upregulated gene in *IGF-1R*^*KO−tdTomato*^ microglia, together with *Aspm*, which is involved in the regulation of the cell cycle [108] (Fig. 4B). Gene ontology (GO) enrichment analysis further showed that multiple differentially expressed genes involved in ribosomal biogenesis and functions were also upregulated in microglia from *IGF-1R*^*KO−tdTomato*^ mice compared to controls (Additional file 2: Fig. 2C).

Conversely, RNA analysis of *IGF-1R*^*KO−tdTomato*^ BAMs showed the upregulation of genes involved in IFN-γ signalling (*Gbp10, Tgtp1*), cell activation (*Slamf9*), myeloid cell differentiation (*Hoxa7*), signal transduction (*Stat5, Capn5, Adcy1*), plasma membrane dynamics (*Mtss2*) and lysosomal degradation (*Hgsnat*) (Fig. 4C, D). In parallel, genes involved in intercellular interactions (*Mfap4, Spata13, Jam2*), immunoregulation (*Itgb3, Plxdc2, Siglec-h*), inhibition of apoptosis (*Arhgap24*), disease associated genes (*Cd9*) and cell cycle (*Ccne1*) were downregulated in BAMs from *IGF-1R*^*KO−tdTomato*^ compared to *IGF-1R*^*WT−tdTomato*^ mice (Fig. 4C, D). GO enrichment analysis in BAMs from *IGF-1R*^*KO−tdTomato*^ mice showed decreased RNA processing and splicing and leukocyte migration, while pathways upregulated indicated increased GTPase and Ras-mediated signalling, cellular growth and amoeboidal-like migration (Fig. 4E). Given the observed changes in genes involved in cellular movement, we isolated microglia and BAMs from the CNS of tamoxifen-treated *IGF-1R*^*WT−tdTomato*^ and *IGF-1R*^*KO−tdTomato*^ mice and analysed the surface expression of adhesion molecules such as integrin αL, β2, α4, αM, and selectin ligand PSGL-1 via flow cytometry. Expression of αL could not be detected in neither BAMs nor microglia (Fig. 4F, G), but the integrin was readily detected on control splenocytes (Fig. 4H). While the expression levels of integrin α4, αL and β2 were comparable between IGF-1R^KO^ and IGF-1R^WT^ cells, PSGL-1 appeared significantly downregulated in BAMs but not microglia from *IGF1R*^*KO−tdTomato*^ mice (Fig. 4F, G).

Taken together, IGF-1R deletion from CNS-resident macrophages led to distinct transcriptomic changes in microglia and BAMs but mostly impacted BAM physiology by regulating pathways involved in RNA processing, growth, intracellular signalling and migration. Accordingly, the absence of IGF-1R also led to the decreased surface expression of the adhesion molecule PSGL-1 in BAMs. Overall, these changes during homeostasis might have a significant impact on the role of BAMs/microglia upon CNS pathology.

### IGF-1R deletion in CNS resident microglia/macrophages increases EAE severity

IGF-1 is considered a potential therapeutic agent for the treatment of several CNS pathologies including multiple sclerosis (MS) [3, 12, 68, 73, 101]. However, IGF-1 administration in MS patients [29] and in the EAE model [5, 10, 18, 22, 33, 52, 54, 56, 103] showed no efficacy or contradictory outcomes. The interpretation of these results is difficult because of the expression of IGF-1R on multiple CNS and immune cell types [27].

**Fig. 5:**
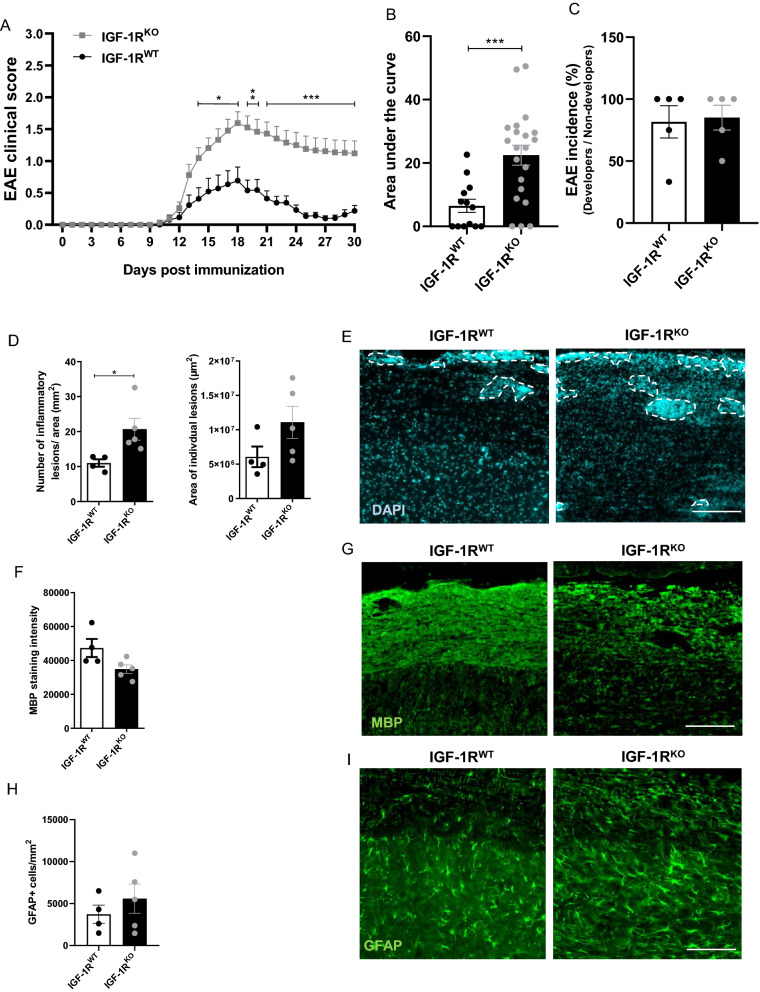
Deletion of IGF1R from CNS-resident myeloid cells leads to worsened clinical signs and tissue inflammation in EAE. EAE was induced in tamoxifen-treated *IGF-1R*^*WT−tdTomato*^ (*n* = 13) and *IGF-1R*^*KO−tdTomato*^ mice (*n* = 21) Shown are: **A** clinical score of disease (mean ± SEM of animals pooled together from 5 independent experiments), statistical analysis: two-way ANOVA, multiple comparisons test of score differences per EAE day between *IGF-1R*^*WT−tdTomato*^ and *IGF-1R*^*KO−tdTomato*^ mice. **B** area under the curve (AUC) (Mann Whitney U test, *p* = 0.001) and **C)** disease incidence in the immunized mice (Mann Whitney U test, *p* = 0.999). Data is represented as mean ± SEM. **D** At the symptomatic peak of disease, CNS tissue was isolated from *IGF-1R*^*WT−tdTomato*^ (*n* = 4) and *IGF-1R*^*KO−tdTomato*^ mice (*n* = 5) and DAPI staining was performed to assess lesion density (number of inflammatory lesions/cm^2^, unpaired t test with Welch`s correction, *p* = 0.0335) and Area of individual lesions (μm^2^). Per mouse, four to five regions have been analysed and the mean was calculated. Each dot represents mean values obtained per mouse. Data is represented as mean ± SEM. (**E**) Representative image displaying spinal cord lesions in *1R*^*WT−tdTomato*^ and *IGF-1R*^*KO−tdTomato*^ mice at peak, white dotted lines indicate single lesions, scale bar 100 μm. (**F**) Following immunostaining against MBP, the degree of demyelination was quantified, displayed is MBP relative staining intensity. Data is represented as mean ± SEM, ). Representative images displaying MBP + staining of spinal cords at EAE peak in *IGF-1R*^*WT−tdTomato*^ and *IGF-1R*^*KO−tdTomato*^*,* scale bar 100 μm. **H** Similarly, after immunostaining against the astrocyte marker GFAP, we quantified the degree of astrogliosis (displayed as GFAP + cell density—cells/mm^2^) **I** Representative pictures of GFAP + staining in spinal cords at EAE peak in *IGF-1R*^*WT−tdTomato*^ and *IGF-1R*^*KO−tdTomato*^*,* scale bar 100 μm. Asterisks indicate significant differences (∗ *p* < 0.05, ∗  ∗ *p* < 0.01 and ∗  ∗  ∗ *p* < 0.001, ∗  ∗  ∗  ∗ *p* < 0.0001)

To understand the role of IGF-1R signalling in CNS-resident macrophages during autoimmune CNS inflammation, we thus induced EAE in tamoxifen-treated *IGF-1R*^*WT−tdTomato*^ and *IGF-1R*^*KO−tdTomato*^ mice. Notably, *IGF-1R*^*KO−tdTomato*^ mice showed a substantially worsened clinical course compared to control mice, with an increased area under the curve and a clinical score that appeared significantly higher than in control mice as soon as 3 days after clinical disease onset and throughout the subsequent symptomatic peak and chronic EAE stages (Fig. 5A, B). Disease incidence was however comparable between experimental groups, thus suggesting that IGF-1R absence did not affect peripheral development of autoimmunity (Fig. 5C). Reflecting the worsened clinical course in *IGF-1R*^*KO−tdTomato*^ mice, we observed significantly higher number and a trend toward an increased size of inflammatory lesions in the inflamed spinal cord of *IGF-1R*^*KO−tdTomato*^ compared to *IGF-1R*^*WT−tdTomato*^ controls at EAE peak (Fig. 5D, E). Furthermore, immunostaining against the myelin protein MBP and the astrocyte marker GFAP in tissue sections from *IGF-1R*^*KO−tdTomato*^ and *IGF-1R*^*WT−tdTomato*^ mice revealed a trend toward increased demyelination and astrogliosis in absence of IGF-1R (Fig. 5F-I).

To further characterize the functional impact of IGF-1R deletion on myeloid cells during inflammation, we quantified the total number of CNS-resident and infiltrating macrophages and assessed differences in their functional phenotype via flow cytometry in *IGF-1R*^*KO−tdTomato*^ vs *IGF-1R*^*WT−tdTomato*^ mice during EAE (Additional file 3: Fig. 3A). At the symptomatic peak of EAE the total number of microglial cells and the expression levels of MHC-II were comparable between experimental groups (Additional file 3: Fig. 3B). Furthermore, despite a statistically non-significant increase in the number of BAMs and infiltrating Ly6C^+^ monocyte-derived cells in the CNS of *IGF-1R*^*KO−tdTomato*^ mice, no significant differences were detected in expression of the phenotypic markers MHC-II, CD206 and CD44 (Additional file 3: Fig. 3C, D). Flow cytometric analysis at the chronic stage of EAE yielded comparable results (data not shown).

In summary, IGF-1R deletion from the majority of CNS-resident macrophages resulted into a significantly stronger clinical development of anti-myelin immunity in the EAE model.

## Discussion

IGF-1 is an essential component of the somatotropic axis regulating body growth [63]. While its systemic ablation leads to aberrant postnatal organ development [100], mice carrying deletion of its receptor IGF-1R die embryonically [53]. In humans, *IGF1R* haploinsufficiency is associated with impaired growth [71]. Notably, beside its systemic hormonal function, IGF-1 signalling acts as an organ-specific modulator of physiological cellular processes [27].

In the CNS, IGF-1 is locally secreted by microglia and BAMs [92] and in parallel is imported to the cerebrospinal fluid (CSF) from the bloodstream, with CSF levels of this growth factor regulated by the concentration of IGF-1 in the serum and by active import through neurovascular coupling [15, 69, 102]. Low levels of IGF-1 in the CSF have been associated with the development of autism-spectrum disorders [107], thus indicating a key physiological role of this molecule at CNS interfaces. Accordingly, while the parenchymal expression of IGF-1R substantially decreases following CNS development [27], IGF-1R expression in resident cells at the border areas of the CNS remains unchanged throughout adulthood [80]. However, the autocrine/paracrine action of this molecule in regulating the physiology of IGF-1-producing BAMs and microglia remains surprisingly undetermined. Together, this missing information hampers our understanding of IGF-1 functions in the CNS and the potential therapeutic use of IGF-1 upon tissue damage.

To shed light on the role of IGF-1R signalling in CNS-resident myeloid cells, we here made use of a novel mouse model (*IGF-1R*^*KO−tdTomato*^) in which *Igf1r* was conditionally deleted in BAMs and microglia. Even though IGF-1R deletion targeted only 68% of microglia and 75% for BAMs, receptor ablation resulted in significant changes at a cellular and, surprisingly, systemic level. More specifically, 5 weeks after recombination, *IGF-1R*^*KO−tdTomato*^ mice showed a significant increase in body weight compared to *IGF-1R*^*WT−tdTomato*^ controls. Homozygous *Igf1r* ablation in hypothalamic neurons or hemizygous *Igf1r* deficiency in neuronal and glial cells were previously reported to influence body mass, leading to a decrease in weight [1, 46], however no reports of growth changes upon myeloid-cell specific mutations in IGF-1R exist. It remains unclear whether the observed effect could be attributed to the deletion of IGF-1R from microglia and BAMs, potentially affecting IGF-1 sensing in pituitary and hypothalamic neurons participating in the somatotropic axis [63], or whether other CX3CR1^+^ macrophages, potentially targeted by our ablation approach in peripheral organs, contributed to the increase in body mass.

Beside these systemic changes, deletion of IGF-1R massively impacted the physiology of BAMs and microglia. Analysis of tissue sections and intravital imaging revealed that both leptomeningeal spinal cord BAMs and brain microglia in *IGF-1R*^*KO−tdTomato*^ mice displayed more complex ramification with higher degree of branches, endpoints and junctions per cell compared to cells from *IGF-1R*^*WT−tdTomato*^ mice. While the correlation between morphological changes and functional alterations in BAMs remains unclear, highly ramified microglia are normally associated with increased homeostatic surveillance and neuroprotective functions [81], with the complexity of microglia ramification regulated by the sensing of neuronal activity and extracellular nucleotides [59]. This is opposed to the “hypertrophic” appearance of these cells upon activation [81] or to “dystrophic” cells observed during ageing and Alzheimer’s disease [28]. Parallel analysis of cell ramifications in the spinal cord did not reveal significant differences between IGF-1R^KO^ and IGF-1R^WT^ microglia but indicated a higher radius of cells from *IGF-1R*^*KO−tdTomato*^ mice – similar to brain microglia- suggestive of increased homeostatic surveillance [26, 75]. In parallel, however, spinal cord microglia from *IGF-1R*^*KO−tdTomato*^ mice showed decreased lacunarity, *i.e.* rotational variation of these cells compared to their counterparts in *IGF-1R*^*WT−tdTomato*^ animals. While the functional meaning of this parameter is context-dependent [48], decreased lacunarity in microglia was observed following IL-1β stimulation [26]. Furthermore, IGF-1R^KO^ microglia in the spinal cord showed a higher density (*i.e.* process thickness) again similar to cytokine-stimulated microglia [26]. In summary, morphological analysis revealed significant differences between BAMs and microglia in *IGF-1R*^*KO−tdTomato*^ and *IGF-1R*^*WT−tdTomato*^ mice but could not provide univocal indications about the differential activation of these cells in situ.

In parallel, transcriptomic analysis revealed that microglia from *IGF-1R*^*KO−tdTomato*^ mice increased expression of *Aspm*, a positive regulator of the cell cycle [11] and *Atp7b*, a gene involved in cellular activation during Alzheimer´s disease [110]. Accordingly, GO analysis indicated increased ribosomal biogenesis, a process typically characterizing microglia activation *e.g.* in response to LPS stimulation [87]. Overall, these changes suggested an augmented basal activation of microglia following IGF-1R deletion.

When analysing the transcriptome of BAMs, several key changes could be observed between IGF-1R^KO^ and IGF-1R^WT^ cells. Firstly, upregulation of genes downstream of IFN-γ signalling (*Gbp10, Tgtp1*) and downregulation of genes involved in immunoregulation (*Itgb3, Plxdc2, Siglec-h*) [37, 84, 91] implied alteration of immune functions in IGF-1R^KO^ BAMs compared to control.

Secondly, pathways linked to metabolic activation were altered in the absence of IGF-1R, and more specifically pathways governing catabolic processes and mRNA processing were downregulated while cell growth processes were upregulated in IGF-1R^KO^ cells from *IGF-1R*^*KO−tdTomato*^ mice. Accordingly, IGF-1R signalling is described as regulator of cellular metabolism in the CNS [27].

Lastly, IGF-1R absence from BAMs affected cellular adhesion and migratory properties, as shown by the increased expression of *Slamf9* [24], the decreased expression of genes involved in intercellular interactions (*Mfap4, Spata13, Jam2*) and decreased “leukocyte migration” process in the GO enrichment analysis from *IGF-1R*^*KO−tdTomato*^ mice. Along the same line, GTPase signalling processes were upregulated in IGF-1R^KO^ cells from *IGF-1R*^*KO−tdTomato*^ mice. This change might be linked to the function of IGF-1R as hybrid receptor able to activate G-protein regulated signalling [17], the latter mediating the regulatory effects of IGF-1 on cell adhesion and migration [16]. Furthermore, again suggesting altered adhesion and migratory capability of IGF-1R^KO^ cells, BAMs from *IGF-1R*^*KO−tdTomato*^ mice showed lower surface expression of PSGL-1, a molecule involved in leukocyte migration with immunomodulatory signalling functions [94].

Taken together, morphological and transcriptomic analysis of CNS-resident myeloid cells upon IGF-1R deletion indicated complex variations of microglia/BAM physiology affecting migration, adhesion, metabolic activation and immune response in these cells.

Not surprisingly, these extensive functional alterations profoundly impacted the development of autoimmune CNS inflammation, with *IGF-1R*^*KO−tdTomato*^ mice showing a significant worsening of clinical EAE progression and of tissue inflammation compared to controls. CNS-resident macrophages exert both protective and detrimental roles during autoimmune neuroinflammation similar to tissue-invading monocyte-derived macrophages [44, 55, 89]. BAMs and microglia become activated even before the development of clinical signs [6, 20] and secrete inflammatory cytokines and chemokines recruiting peripheral immune cells into the CSF [79, 83]. The observed clinical worsening of EAE, also reflected by increased density of inflammatory lesions in *IGF-1R*^*KO−tdTomato*^ mice, might thus suggest that IGF-1R signalling in BAMs/microglia exert a significant protective role limiting the development of CNS autoimmunity. While the mechanism underlying this amelioration remain unclear, the comparable number and expression of activation markers in CNS myeloid cells from *IGF-1R*^*KO−tdTomato*^ compared to *IGF-1R*^*WT−tdTomato*^ mice suggest that this disease-ameliorating effect is mostly mediated via an indirect modulation of the CNS environment by microglia/macrophages upon IGF-1R deletion.

## Conclusions

Our study in the *IGF1R*^*KO−tdTomato*^ model shed light for the first time on the role of IGF-1 signalling in CNS-resident macrophages. We here showed that loss of IGF-1R in these cells leads to: (I) altered cellular morphology; (II) regulation of cellular transcriptome affecting adhesive, metabolic and immune functions; (III) worsened autoimmune CNS inflammation. Furthermore, even though the observed changes might be affected by cellular heterogeneity and by the differential efficiency in receptor deletion, our work suggests differences in the role of IGF-1R between BAMs and microglia, with microglia phenotypically affected by absence of the receptor and BAMs displaying broader transcriptomic changes upon *Igf1r* deletion. BAMs are strategically located at CNS interfaces and extensively interact with invading immune cells during pathology [30, 45, 109]. Notably, since we observed a massive impact on the evolution of neuroinflammation upon a genetic modification affecting only 70% of BAMs, our model suggests a central role of these cells in inflammatory responses within the CNS.

Taken together, given the results obtained in our deletion model, it is tempting to speculate that IGF-1R in CNS-resident macrophages play a tonic anti-inflammatory function similar to the one described in peripheral macrophages [32, 41, 50, 88]. Further experiments are however needed to corroborate this hypothesis.

## Supplementary Information

**Additional file 1.** Morphological characterization of CNS-resident myeloid cells following ablation of IGF-1R.To assess the morphology of CNS resident microglia (brain and spinal cord) and leptomeningeal BAMs in IGF-1RKO-tdTomato (n=4 mice) compared to IGF-1RWT-tdTomato (n=4 mice), we performed Sholl analysis, skeletal analysis and fractal-lacunarity analysis (see methods for more details). The parameters obtained from these 3 morphological analyses were grouped into 4 categories indicating cell size (comprising quantification of soma size, cell density, critical value defined as distance from soma where maximum number of branches occurred), cell shape/elongation (characterized by span ratio and circularity), cell complexity (quantification of cell lacunarity and maximum cell span across a convex hull) and cell ramification (illustrated through summed branch length/cell, maximum branch length/ cell, maximum intersections/cell, number of endpoints/cell (measurement of cell contact with environment), number of junctions/ cell). Number of cells analysed: Brain Microglia: IGF-1RKO-tdTomato (n=84), IGF-1RWT-tdTomato (n=71); Spinal Cord Microglia: IGF-1RKO-tdTomato (n=75) IGF-1RWT-tdTomato (n=66); Leptomeningeal BAMs: IGF-1RKO-tdTomato (n=78) IGF-1RWT-tdTomato (n=91). All values are presented as mean ± SEM. Statistical analysis was performed by using unpaired t test with Welch’s correction for normally distributed data or Mann-Whitney U test for non-normally distributed data. Asterisks indicate significant differences (∗p < 0.05, ∗∗p < 0.01 and ∗∗∗p < 0.001, ∗∗∗∗p < 0.0001).

**Additional file 2.** Transcriptomic changes in microglia and BAMs upon IGF-1R deletion. A) Sorting strategy of microglia (CD45+CD11b+P2ry12+tdTomato+) and BAMs (CD45+CD11b+P2ry12-CD206+tdTomato+). Cells were sorted from the brain of IGF-1RKO-tdTomato and IGF-1RWT-tdTomato mice directly into RNA protect buffer. B) Venn diagram showing the concordance of gene expression differences in microglia and BAMs from IGF-1RKO-tdTomato and IGF-1RWT-tdTomato mice C) Functional enrichment analysis of the microglia from IGF-1RKO-tdTomato and IGF-1RWT-tdTomato mice using all pathways from the Gene Ontology database. Left, pathways upregulated in IGF-1RKO-tdTomato mice; right, pathways downregulated in IGF-1RKO-tdTomato mice.

**Additional file 3.** Flow cytometry characterization of CNS-myeloid cell number and phenotype at the symptomatic peak of EAE. A) Gating Strategy for analysis of CNS resident myeloid cells isolated from brain and spinal cords including leptomeninges of IGF-1RKO-tdTomato and IGF-1RWT-tdTomato mice at EAE peak (clinical manifestation: hind leg paralysis). Cells were identified based on their size and granularity, using the forward versus side scatter gating (FSC vs SSC). Following single cell gating (FSC-H vs FSC-A) and selection of alive cells (live-dead staining), we gated for CD45 vs CD11b expression and analyzed two distinct cell populations: CD45highCD11b+ cells and CD45intermediateCD11b+ cells. Within the CD45highCD11b+ cell population, we further gated on Tomato positivity, thus identifying CNS-resident CD45highCD11b+Tomato+ BAMs (C) and CD45highCD11b+Tomatoneg cells illustrating CNS infiltrating blood-derived myeloid cells. The latter population was further gated for Ly6G vs Ly6C positivity and CD45highCD11b+TomatonegLy6GnegLy6C+ population representing CNS infiltrating monocyte-derived macrophages (D) was selected for the final analysis. CNS resident microglial cell population was identified based on CD45intermediateCD11b+Tomato+ expression (B). Representative histograms of marker expression profiles of MHCII, CD44, CD206 on respective myeloid cell populations analysed. We display the absolute number of CNS resident microglial cells (D), BAMs (E) and CNS-infiltrating monocyte-derived macrophages (C) in IGF-1RKO-tdTomato (n=4) and IGF-1RWT-tdTomato (n=4) mice at EAE peak and relative expression (mean fluorescence intensity of sample relative to isotype control staining) or percentage of positive cells of MHC-II, CD206, CD44 molecules. No statistically significant differences were observed in either of the parameters checked within the three populations of cells investigated between in IGF-1RKO-tdTomato and IGF-1RWT-tdTomato mice. All values are presented as mean ± SEM. Statistical analysis was performed by using unpaired t test with Welch`s correction.

## Data Availability

The data that support the findings of this study are available from the corresponding author upon reasonable request.

## Abbreviations

CNS: Central nervous system
MS: Multiple sclerosis
EAE: Experimental autoimmune encephalomyelitis
BAM: Barrier-associated macrophage
IGF-1: Insulin-like growth factor 1
IGF-1R: Insulin-like growth factor 1 receptor
