# Supplementary figures and images for: Insulin-like growth factor-1 receptor controls the function of CNS-resident macrophages and their contribution to neuroinflammation
Source: Acta Neuropathol Commun. 2023 Mar 8;11:35. doi: 10.1186/s40478-023-01535-8 (PMC9993619; doi:10.1186/s40478-023-01535-8)

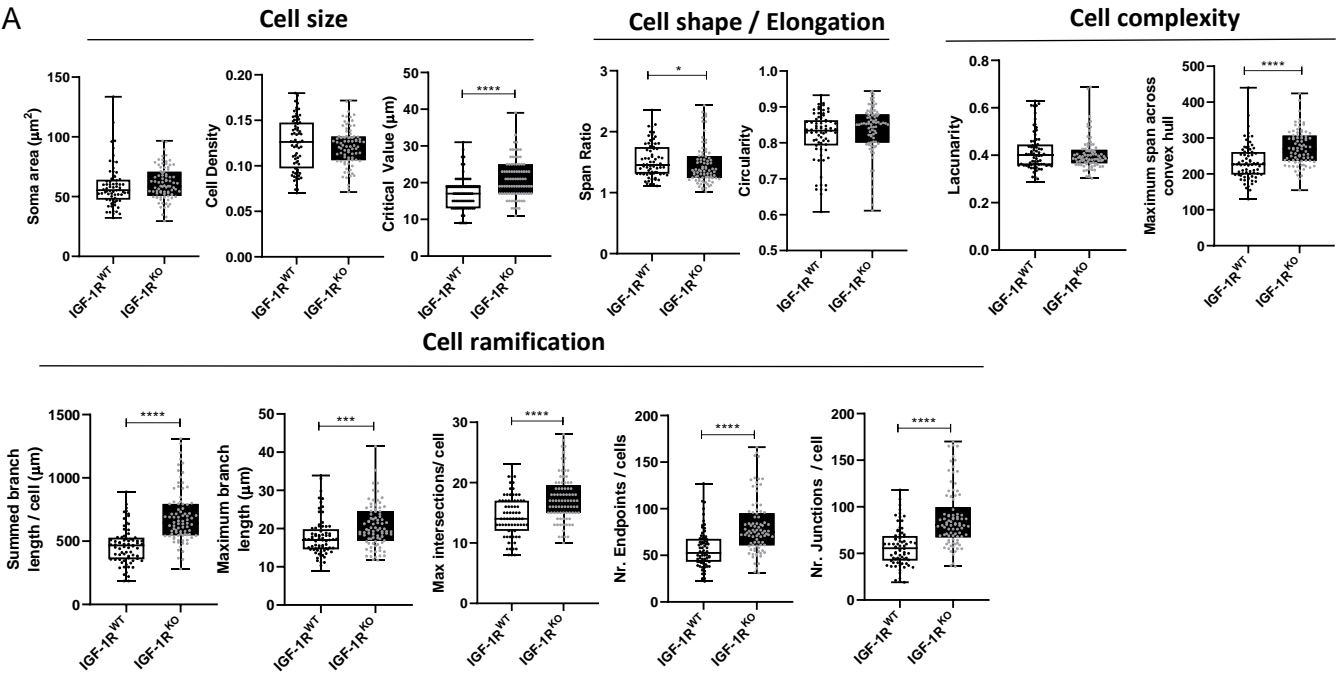

Microglia (Spinal Cord)

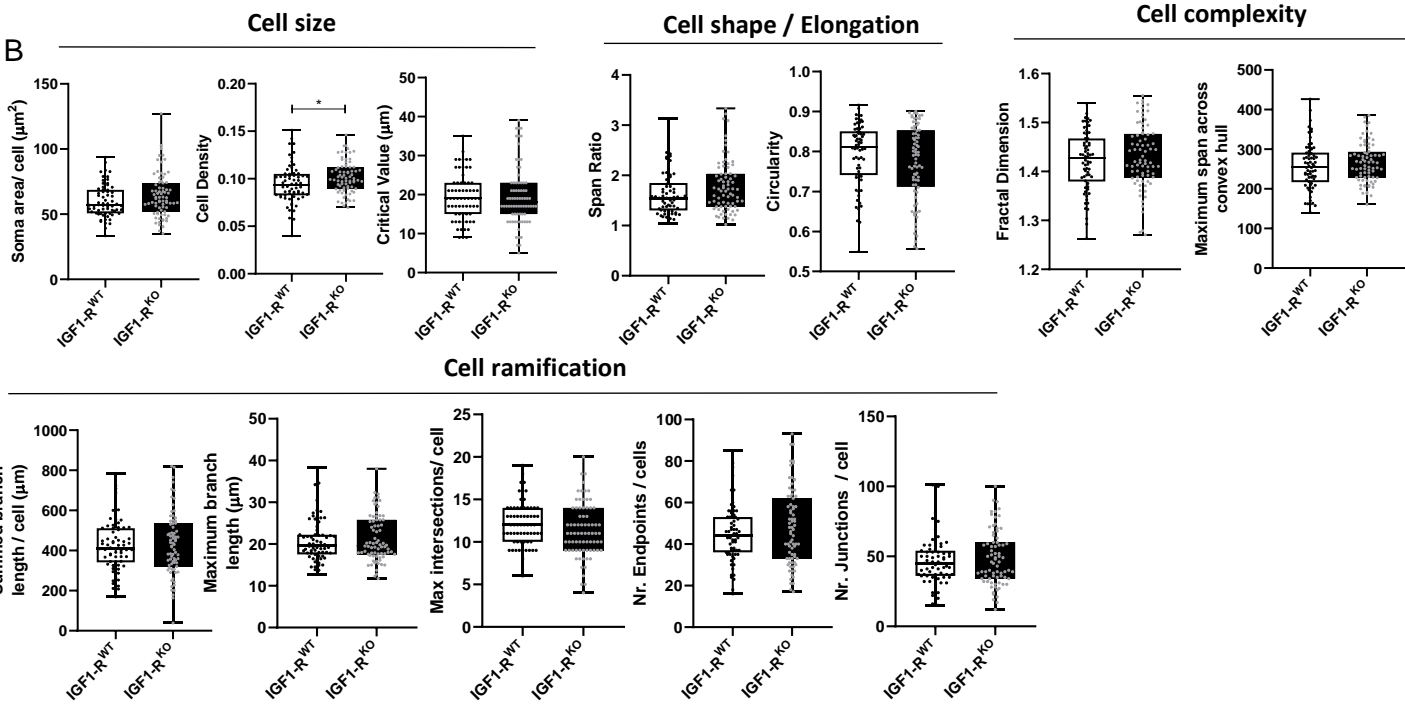

BAMs (leptomeninges)

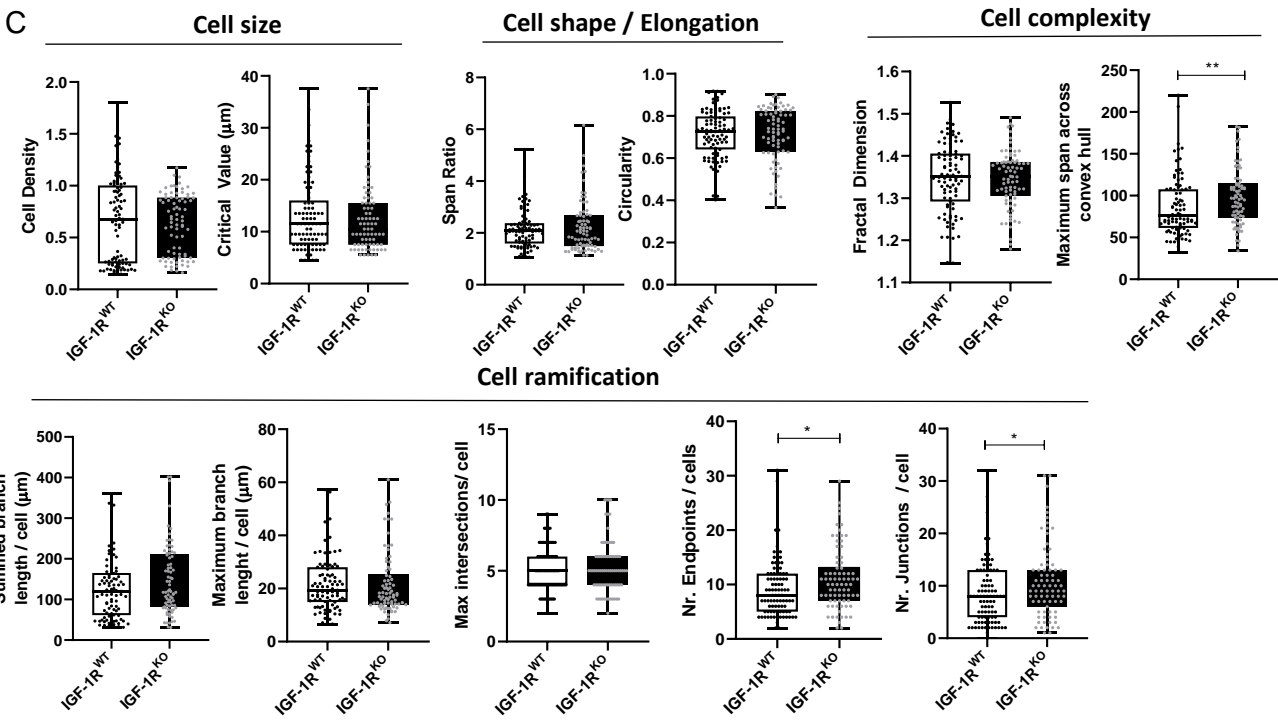

Supplement: Supplementary file 1 — Additional file 1. Morphological characterization of CNS-resident myeloid cells following ablation of IGF-1R.To assess the morphology of CNS resident microglia (brain and spinal cord) and leptomeningeal BAMs in IGF-1RKO-tdTomato (n=4 mice) compared to IGF-1RWT-tdTomato (n=4 mice), we performed Sholl analysis, skeletal analysis and fractal-lacunarity analysis (see methods for more details). The parameters obtained from these 3 morphological analyses were grouped into 4 categories indicating cell size (comprising quantification of soma size, cell density, critical value defined as distance from soma where maximum number of branches occurred), cell shape/elongation (characterized by span ratio and circularity), cell complexity (quantification of cell lacunarity and maximum cell span across a convex hull) and cell ramification (illustrated through summed branch length/cell, maximum branch length/ cell, maximum intersections/cell, number of endpoints/cell (measurement of cell contact with environment), number of junctions/ cell). Number of cells analysed: Brain Microglia: IGF-1RKO-tdTomato (n=84), IGF-1RWT-tdTomato (n=71); Spinal Cord Microglia: IGF-1RKO-tdTomato (n=75) IGF-1RWT-tdTomato (n=66); Leptomeningeal BAMs: IGF-1RKO-tdTomato (n=78) IGF-1RWT-tdTomato (n=91). All values are presented as mean ± SEM. Statistical analysis was performed by using unpaired t test with Welch’s correction for normally distributed data or Mann-Whitney U test for non-normally distributed data. Asterisks indicate significant differences (∗p < 0.05, ∗∗p < 0.01 and ∗∗∗p < 0.001, ∗∗∗∗p < 0.0001). [file 40478_2023_1535_MOESM1_ESM.pdf]

SUPP FIG.2

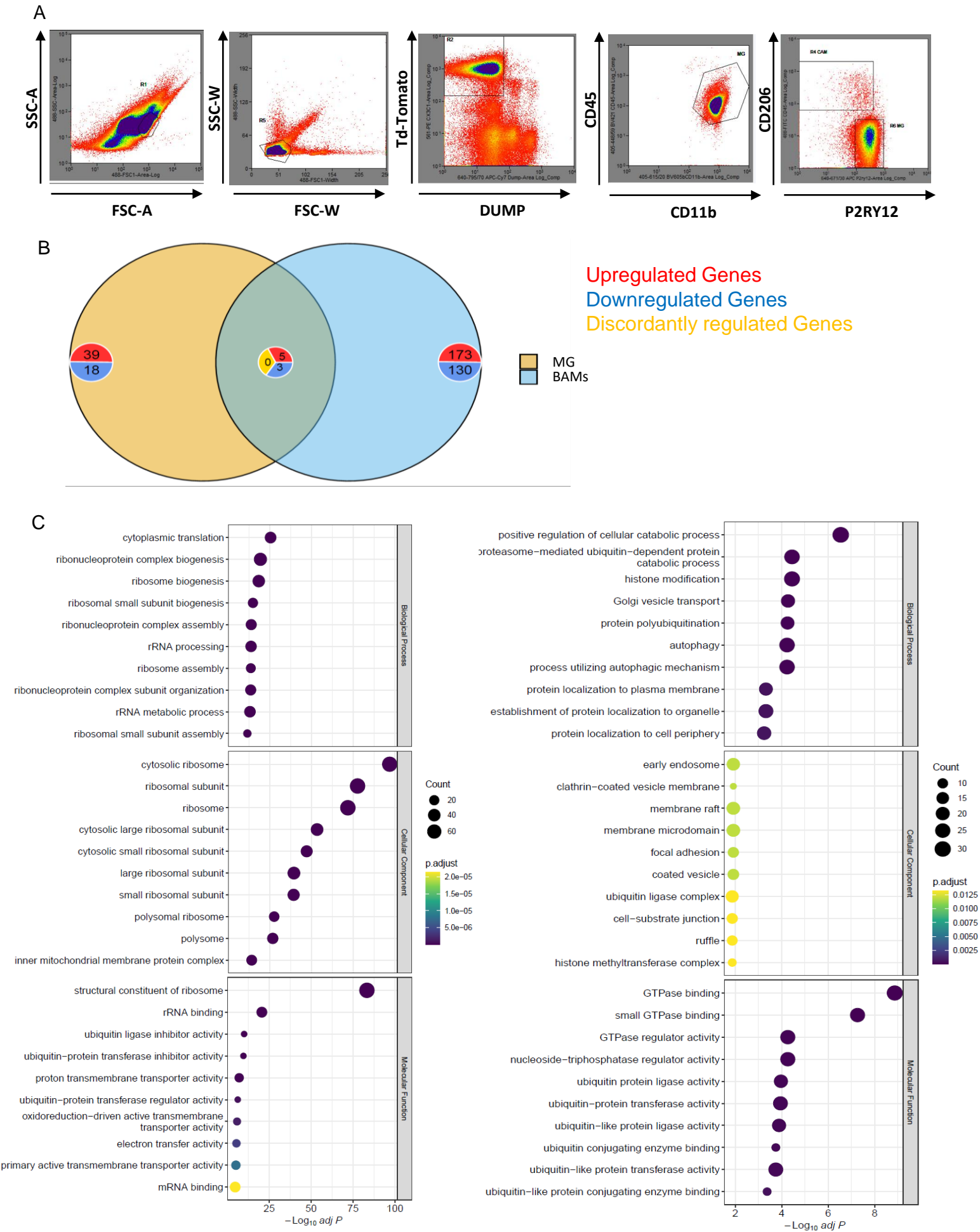

Supplement: Supplementary file 2 — Additional file 2. Transcriptomic changes in microglia and BAMs upon IGF-1R deletion. A) Sorting strategy of microglia (CD45+CD11b+P2ry12+tdTomato+) and BAMs (CD45+CD11b+P2ry12-CD206+tdTomato+). Cells were sorted from the brain of IGF-1RKO-tdTomato and IGF-1RWT-tdTomato mice directly into RNA protect buffer. B) Venn diagram showing the concordance of gene expression differences in microglia and BAMs from IGF-1RKO-tdTomato and IGF-1RWT-tdTomato mice C) Functional enrichment analysis of the microglia from IGF-1RKO-tdTomato and IGF-1RWT-tdTomato mice using all pathways from the Gene Ontology database. Left, pathways upregulated in IGF-1RKO-tdTomato mice; right, pathways downregulated in IGF-1RKO-tdTomato mice. [file 40478_2023_1535_MOESM2_ESM.pdf]

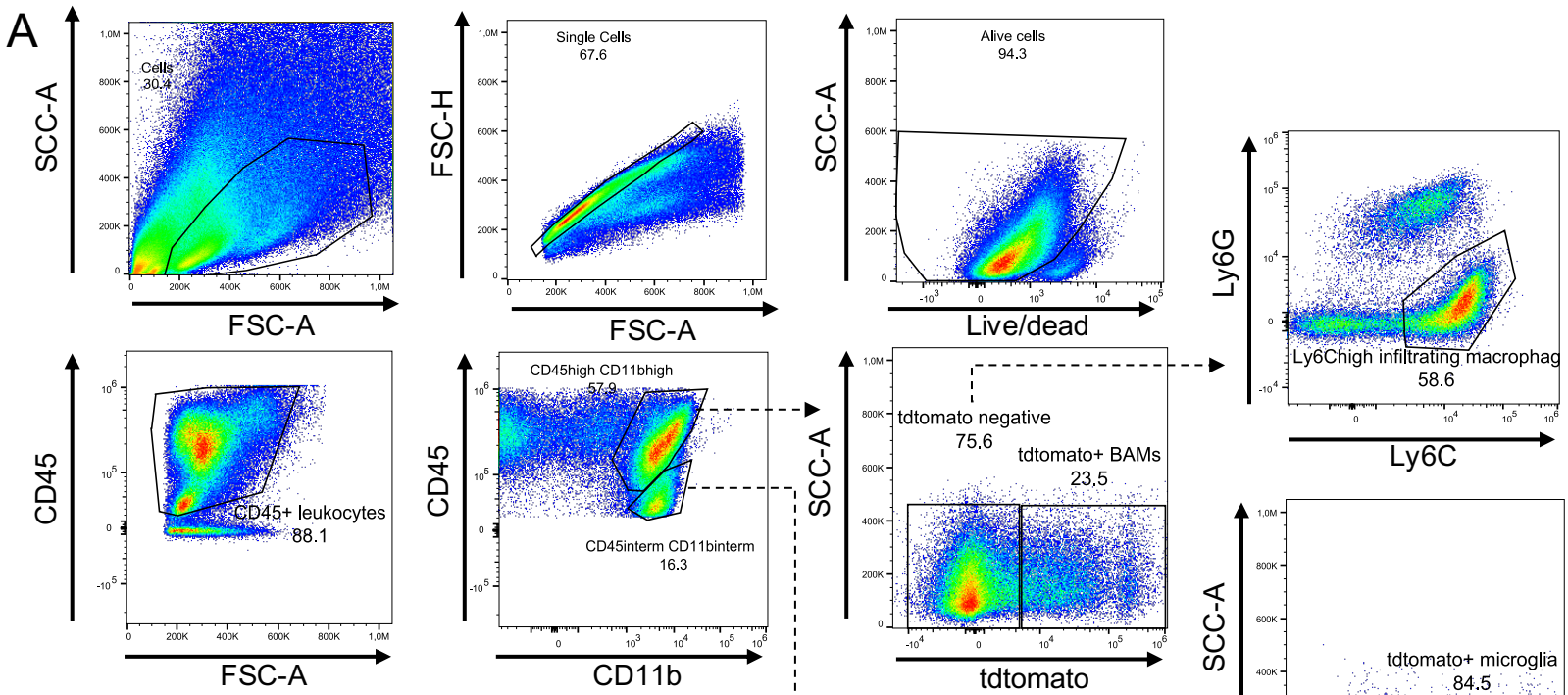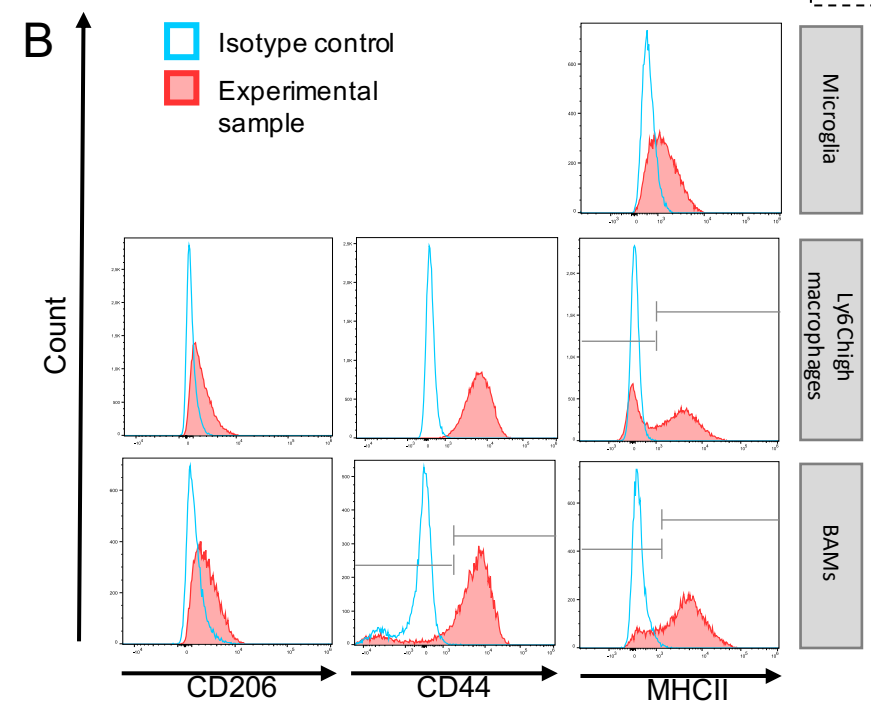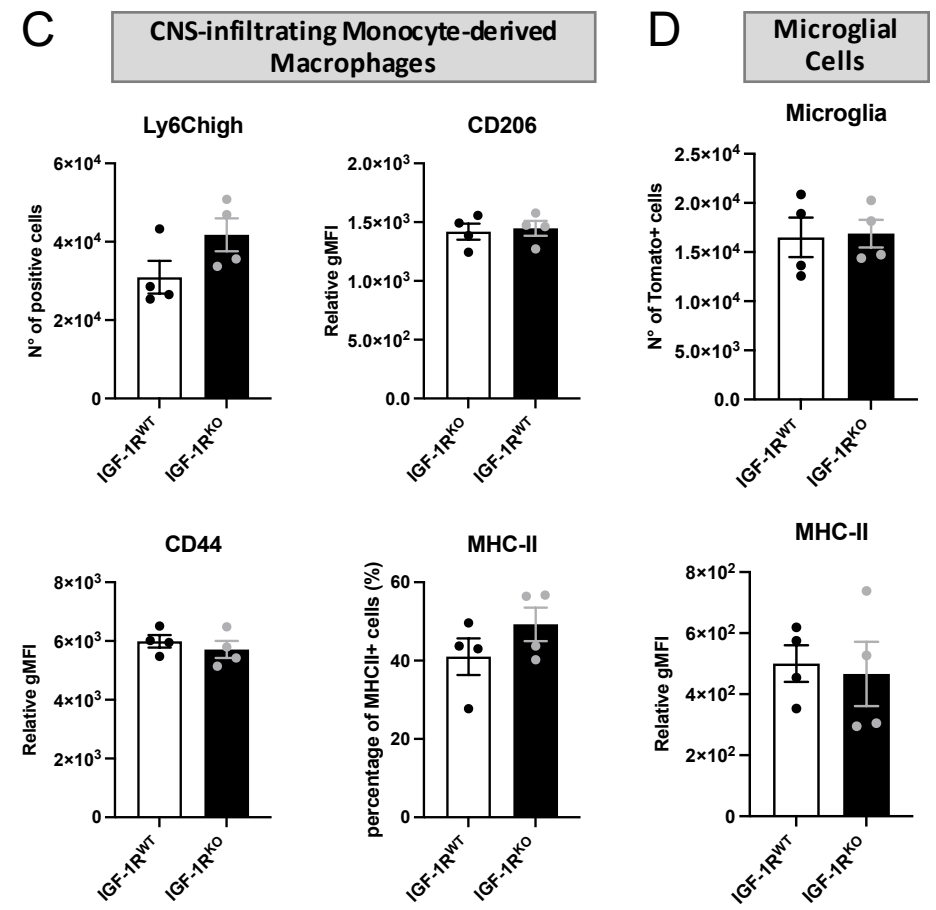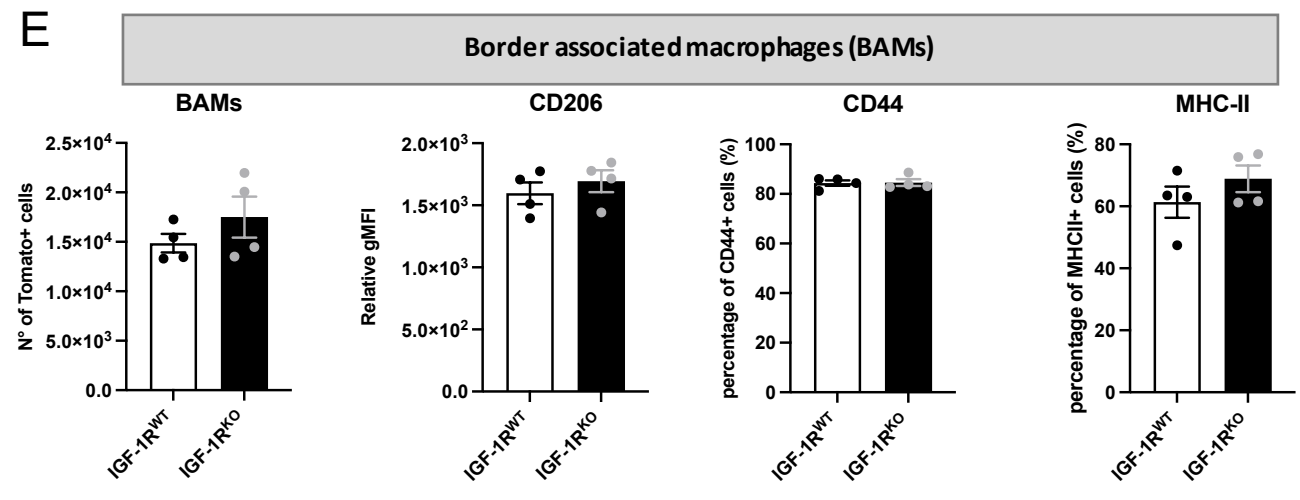

Supplement: Supplementary file 3 — Additional file 3. Flow cytometry characterization of CNS-myeloid cell number and phenotype at the symptomatic peak of EAE. A) Gating Strategy for analysis of CNS resident myeloid cells isolated from brain and spinal cords including leptomeninges of IGF-1RKO-tdTomato and IGF-1RWT-tdTomato mice at EAE peak (clinical manifestation: hind leg paralysis). Cells were identified based on their size and granularity, using the forward versus side scatter gating (FSC vs SSC). Following single cell gating (FSC-H vs FSC-A) and selection of alive cells (live-dead staining), we gated for CD45 vs CD11b expression and analyzed two distinct cell populations: CD45highCD11b+ cells and CD45intermediateCD11b+ cells. Within the CD45highCD11b+ cell population, we further gated on Tomato positivity, thus identifying CNS-resident CD45highCD11b+Tomato+ BAMs (C) and CD45highCD11b+Tomatoneg cells illustrating CNS infiltrating blood-derived myeloid cells. The latter population was further gated for Ly6G vs Ly6C positivity and CD45highCD11b+TomatonegLy6GnegLy6C+ population representing CNS infiltrating monocyte-derived macrophages (D) was selected for the final analysis. CNS resident microglial cell population was identified based on CD45intermediateCD11b+Tomato+ expression (B). Representative histograms of marker expression profiles of MHCII, CD44, CD206 on respective myeloid cell populations analysed. We display the absolute number of CNS resident microglial cells (D), BAMs (E) and CNS-infiltrating monocyte-derived macrophages (C) in IGF-1RKO-tdTomato (n=4) and IGF-1RWT-tdTomato (n=4) mice at EAE peak and relative expression (mean fluorescence intensity of sample relative to isotype control staining) or percentage of positive cells of MHC-II, CD206, CD44 molecules. No statistically significant differences were observed in either of the parameters checked within the three populations of cells investigated between in IGF-1RKO-tdTomato and IGF-1RWT-tdTomato mice. All values are presented as mean [file 40478_2023_1535_MOESM3_ESM.pdf]
